# Integrating competency-based, interprofessional teamwork education for students: guiding principles to support current needs and future directions

**DOI:** 10.3389/fmed.2024.1490282

**Published:** 2025-01-07

**Authors:** Kimberly N. Williams, Elizabeth H. Lazzara, Jessica Hernandez, David Klocko, Neethu Chandran, Shannon L. Paquette, Richard Preble, Mozhdeh Sadighi, Bau Tran, Molly Kilcullen, Robert Rege, Gary Reed, Eduardo Salas, Scott I. Tannenbaum, Philip E. Greilich

**Affiliations:** ^1^Department of Human Factors and Behavioral Neurobiology, Embry-Riddle Aeronautical University, Daytona Beach, FL, United States; ^2^Department of Emergency Medicine, UT Southwestern Medical Center, Dallas, TX, United States; ^3^Department of Physician Assistant Studies, UT Southwestern Medical Center, Dallas, TX, United States; ^4^Department of Anesthesiology and Pain Management, UT Southwestern Medical Center, Dallas, TX, United States; ^5^Office of Undergraduate Medical Education, UT Southwestern Medical Center, Dallas, TX, United States; ^6^Department of Psychological Sciences, Rice University, Houston, TX, United States; ^7^Department of Surgery, Office of Undergraduate Medical Education, UT Southwestern Medical Center, Dallas, TX, United States; ^8^Department of Internal Medicine, Office of Quality, Safety and Outcomes Education, UT Southwestern Medical Center, Dallas, TX, United States; ^9^The Group for Organizational Effectiveness, Inc., Albany, NY, United States; ^10^Department of Anesthesiology and Pain Management, Offices of the Undergraduate Medical Education and Quality, Safety and Outcomes Education, UT Southwestern Medical Center, Dallas, TX, United States

**Keywords:** teamwork training, interprofessional, competency-based medical education, healthcare education, curriculum design, implementation, longitudinal assessment

## Abstract

Interprofessional teamwork is vital to effective patient care, and targeting healthcare learners earlier in their education can lead to greater improvement in confidence and competence in teamwork skills. Despite this, institutions have continued struggling to integrate competency-based interprofessional teamwork curriculum in undergraduate health care professions’ education. The current article provides guidance related to design, implementation, and assessment for institutions seeking to implement competency-based teamwork education and training strategies for healthcare students. Guiding principles and strategies for curricular design focus on conducting thorough interprofessional needs analyses and building transportable, evidence-based competencies that apply across professions. For implementation, key principles center on strategies to ensure adequate professional representation and faculty development. Assessment considerations focus on building infrastructure for evaluation that spans professional schools. These strategies aim to create a robust, effective, and sustainable IPE curriculum that enhances collaboration and teamwork among future healthcare professionals. By addressing the key areas of design, implementation, and assessment, this article offers comprehensive guidelines for advancing interprofessional education. We believe incorporating the key guiding principles and strategies from this paper will enable institutions to integrate teamwork education and training more effectively into undergraduate healthcare training, which will facilitate institutions’ ability to ensure learners are “team ready” as they transition into the workforce after graduation.

## Introduction

It is well established that teamwork is vital for providing safe and effective patient care. Healthcare students have the capacity to impact patient care through their interactions on teams and with patients even while in training. Residents may be particularly vulnerable to committing preventable errors if teamwork skills are lacking, which can negatively impact patient care (1). Given their direct role in patient care, there has been a recent shift in viewing residents more as providers than as trainees (2). This requires healthcare students to be “team ready” upon graduation from their pre-licensure programs. This shift is supported by the American Association of Medical Colleges (AAMC) specifying teamwork competencies needed for the transition to residency through its Entrustable Professional Activities (EPAs) (3). Similarly, the Canadian Interprofessional Health Collaborative’s (CIHC) competency framework for advancing collaboration (4) and the Interprofessional Education Collaborative (IPEC) competencies (5) target teamwork and maintain relevance across healthcare professions in the career space. Evidence shows earlier introduction of such competencies confer greater confidence and competence in first year post-graduate residents upon entering residency (6). Further, evidence suggests that biases between healthcare professions are formed early, prior to interprofessional education (IPE) in students (7), which is echoed by current stereotypes held by both students and the public (8, 9). Thus, introducing education in teamwork competencies as early as possible may reduce the need for unlearning of bad habits upon entry into the workforce – in this case about teamwork and interprofessional collaboration. Incorporating longitudinal education and assessment opportunities additionally permits learners to receive extended feedback, see their own progress and its impact on their teams over time (10).

The Center for the Advancement of Interprofessional Education (CAIPE) defines interprofessional education as “occasions when two or more professions learn with, from and about each other to improve collaboration and the quality of care” (11). Although exposure to people from other professions may be beneficial, without some degree of structure, there is the risk of learning incorrect lessons/insights. Therefore, it is relevant to intentionally target these learners for teamwork and interprofessional learning experiences as early as possible to minimize negative repercussions of poor teamwork competencies and/or stereotype biases. Despite this, institutions have struggled to incorporate such curricular events in the undergraduate medical education space (12). The current article provides guidance related to design, implementation, and assessment for institutions seeking to implement competency-based teamwork education and training strategies for healthcare students, which are informed by the literature and our collective experience as healthcare professionals, educators, administrators, and assessment experts collaborating through the TeamFIRST program at University of Texas Southwestern Medical Center. Guiding principles and strategies for curricular design focus on conducting thorough interprofessional needs analyses and building transportable, evidence-based competencies that apply across professions. For implementation, key principles center on strategies to ensure adequate professional representation and faculty development. Assessment considerations focus on building infrastructure for documentation that spans professional schools. We believe incorporating the key guiding principles and strategies from this paper will enable institutions to integrate teamwork education and training more effectively into undergraduate healthcare curriculum, which will facilitate institutions’ ability to ensure learners are “team ready” as they transition into the workforce after graduation.

## Design recommendations: needs analysis and faculty selection

The first step in the process of generating an effective IPE program is to conduct a thorough needs analysis that identifies competencies for longitudinal instruction and assessment which effectively incorporate nuances between professional groups. Although there are existing needs analyses for healthcare [e.g., the Hennessy-Hicks Training Needs Analysis questionnaire; (13)], these often rely on self-report mechanisms to identify training needs. For example, the Hennessy-Hicks questionnaire assesses series of clinical tasks and requests providers to rate (1) how critical the task is for their job and (2) how well they are performing the task. Not only is this method targeted to healthcare providers (rather than trainees), but this method also makes the needs analysis vulnerable to the potential role of survey biases and the Dunning-Kruger effect altering the results of the needs analysis. The Dunning-Kruger effect refers to a cognitive bias where individuals with limited competence in a particular domain tend to overestimate their abilities, while those with higher competence may underestimate theirs (14). To avoid these issues, it would be beneficial for future research to focus on the development of a standardized teamwork training needs analysis targeted to the undergraduate professional education space. Strategies for effective integration of team-oriented needs analyses include card sorting tasks to objectively evaluate shared mental models (15), surveys to evaluate teamwork contexts (16), and simulation to evaluate team performance (17). To maximize generalizability, such needs analyses should be aligned with existing competency frameworks. For example, the Canadian Interprofessional Health collaborative’s (CIHC) competency framework for advancing collaboration (4) specifies six domains of competency that relate to communication and teamwork. These include relationship-focused care services, team communication, role clarification and negotiation, team functioning, team differences/disagreements processing, and collaborative leadership. Similarly, the Interprofessional Education Collaborative (IPEC) competencies include four domains that span values and ethics, roles and responsibilities, communication, and teams and teamwork.

Competency frameworks such as IPEC and CIHC often overlap, and it can be challenging to identify which should take priority when beginning an interprofessional needs assessment. Institutions may find utility in reviewing existing research-based consensus methods that have been used to evaluate IPE competency frameworks thus far and using these as a starting point to begin an institutional consensus evaluation when planning the needs analysis. Rogers et al. (18) conducted two international workshops to come up with international consensus on aspects that are vital to assessment of interprofessional learning in the context of interprofessional education. They identified five domains as key themes relevant across competency frameworks that should be incorporated in assessment: role understanding, interprofessional communication, coordination/collaborative decision-making, interprofessional values, reflexivity, and teamwork. These can be utilized as a baseline for identifying competency frameworks most relevant to an institution’s goals. Initial needs assessments should rely heavily on input from key stakeholders to ensure all aspects of the assessment align with the interests and logistical realities across levels of the organization (19). Thus, it can be helpful to conduct consensus exercises with key stakeholders within the organization to solidify the competency framework and subsequent competencies to focus on during the needs analysis phase. For example, to produce design guidelines for assessment using their competency framework, Smeets et al. (20) compiled separate expert groups consisting of interprofessional experts, patients, educational scientists, students, and teachers. They had each of these groups meet to come to consensus regarding key guidelines for IP assessment plans at their institution regarding three key features: (1) the assessment tasks, (2) the pool of assessors, and (3) procedures that should be used to assess IP competencies in students. They then had meetings with representatives from each of these groups to come to consensus across the different types of stakeholders. This strategy enabled them to reach consensus across stakeholder groups in most of their guidelines for assessment (20). Thus, these methods may be an effective way for organizations to identify the competency framework and individual competencies that should be targeted, and subsequently identify the specific assessment methods that can be used to inform a targeted educational improvement plan at their institution. Benefits and disadvantages of varying data collection methods for needs assessments can be found in Goldstein and Ford (21).

**Figure 1:**
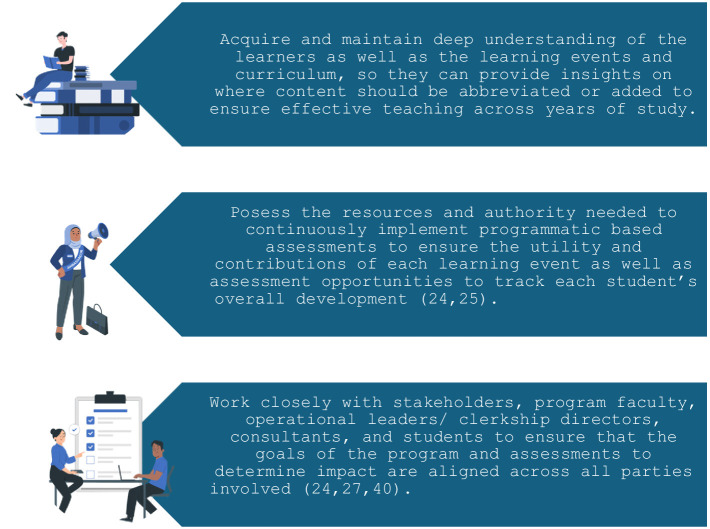
Key abilities of the program’s core faculty. Illustrations provided by StorySet (https://storyset.com/).

Although it is common practice to begin instructional design improvements through needs analyses (22), without core faculty to guide the program and assessment, educational interventions are vulnerable to overlapping with one another and achieving insufficient depth to enable the learner to progress through mastery across the learning objectives in longitudinal curriculums (23, 24). Therefore, establishing a core faculty of educators or champions that take responsibility for maintaining clear and deep learning objectives across the full curriculum is beneficial to ensure appropriate sequencing of performance opportunities, learning events, and content (25). Maximum success for progressive educational interventions and behavioral assessments requires significant involvement from faculty scholars to oversee longitudinal goals of the program (24, 26). It is beneficial to target faculty involved in consensus processes in the needs analysis phase as primary individuals for these roles, as participating in the consensus process enables them to have a greater understanding of the program’s scope than individuals who were not involved in such processes. Key skills, knowledge, and functions of the ideal individual(s) to represent core faculty are outlined in Figure 1. Failure to meet these requirements can ultimately cause substantive conflict between the stakeholders’ needs and the students and faculty at the front line of the program, which lead to wasted effort and threaten program utility and sustainability (27).

## Implementation recommendations: piloting and faculty development

Once the training needs analysis has been completed and core faculty have designed the curriculum, designers should ensure all module plans are appropriately built on each other throughout each iteration and according to the potential for co-education across health professionals. Piloting is vital to the success of this process (28). Pilot sessions create an early opportunity for iteration on developed curriculum, activities, and rubrics, and allow further improvement opportunities when they continue to be implemented in each cohort (28). To get the most out of piloting sessions, they should ideally incorporate a selection of the faculty who will lead the learning activity, as well as students who will participate and/or have participated in a previous event (if the activity has previously proceeded to the implementation stage). Both sets of students add utility to piloting sessions. While former students can use their culminated experience to advise changes that enhance its usefulness to prepare students for subsequent courses or clinical practice, students who are naïve to the intervention can provide a fresh perspective that serves as a more realistic test of how the event will be perceived by the incoming student cohort. As evidence of the benefits of incorporating diverse student opinions in curriculum development, the University of Illinois College of Medicine – Chicago generated the Student Curricular Board (SCB) to leverage students’ expertise for curriculum improvements, and discovered this program was effective in using student knowledge to lead to improvements in program evaluation and longitudinal curriculum design (29). They found participation in the SCB additionally benefited student awareness of program initiatives and increased their inclination to pursue careers in academic medicine (29). Such programs also offer opportunities to further develop faculty representatives who deliver content to students.

When implementing a curriculum at a large academic medical center, it is rarely practical to have few faculty implement all program-related content. Additionally, institutions often face significant barriers in coordinating professional school schedules and balancing student ratios to create an adequate IPE experience (30). The impact of these barriers on IPE can be mitigated by integrating faculty representatives from varying disciplines into IPE to ensure adequate professional representation (8). For example, a UK university implemented their IPE program by having two faculty members (one from each program) deliver content in tandem to model appropriate behaviors to groups of students (8). To meet the need for diverse faculty, early efforts to establish a core faculty should further extend to a representative set of faculty members who deliver series of related learning activities to the students. Development of extended faculty is vital for identifying informal norms within the organization and combating areas where it contradicts the learning objectives (e.g., if in practice some clinicians at the institution do not adhere to the standardized handover protocols being taught or adequately engage in interprofessional collaboration (25)). Through this process, institutions can make developing faculty a priority (25), which further promotes these individuals to become education leaders, champions, and role models. There are a variety of methods to develop faculty. Examples of successful methods include provision of multiple forms of resources to aid teaching sessions (e.g., written and video-based content, Q&A sessions, and technical support), incorporating opportunities to participate in pilot sessions and co-create the curriculum, as well as post-activity debriefing sessions to provide feedback, and attend curriculum-specific educational sessions and workshops to improve their teaching and understanding of teamwork competencies. For example, McMillan et al. (31) describes key strategies to establish faculty commitment and ownership through conducting faculty-led workshops and disseminating data-driven findings to faculty. One of the leading causes of faculty resistance to change in medical education contexts is lack of common vision and consensus (32). Thus, the methods recommended in the previous section to reach consensus on the selected competency framework and needs analysis procedures may be a powerful mechanism to reduce faculty resistance to change during the implementation phase. Consensus meetings may also help to develop groups of faculty members and students representing each discipline and enhance interprofessional experience within these groups, which may further facilitate transfer of training to the clinical environment by contributing to the development of communities of practice that help students and faculty learn within the workplace (33, 34). Further, capitalizing on the experience of faculty involved in the needs analysis stage enables their insights from the needs analysis to transfer into implementation of the curriculum. Such efforts can be instrumental in ensuring that faculty have the knowledge and skills to convey curriculum content to students in ways that are more likely to be reflected in assessments.

## Assessment recommendations: infrastructure and logistics

For assessment, IPE can be greatly aided by developing a capacity for academic evaluation that spans professional schools and facilitates collaboration. Assessing progression to mastery requires longitudinal assessment and linking many sources of data across the learners’ duration in the program (23). Although it is often easier in the short term to allow individuals to fill in names using free-text fields or handwriting, this system is vulnerable to error and highly inefficient in the long term. It is nearly inevitable to have several students within a given course have similar or even identical names, which renders them insufficient for the purpose of longitudinal identification, and nicknames can both help and hinder this process. Linking longitudinal data can be much more successful by establishing a database that links student full names, nicknames, and student ID numbers, and provides these linked pieces of information to raters in a drop-down format across surveys. Tools with survey functionality such as REDCap (35) can be completed using smart phones, tablets, or laptops, making them a reasonably adequate replacement for paper form. For institutions that do not have access to REDCap, Qualtrics™ also has capabilities for longitudinal assessment and data linkages. Each of these systems can be programmed to utilize or download study ID numbers in place of identifiable information if there are substantive privacy concerns (though this is not typically the case, given these systems are secure and may only be accessed by those specified to have access rights). A system that includes multiple sources of identifying information enables raters to easily select the correct student based on a variety of information, rather than any single piece, and standardizes the identification process across assessment methods can greatly improve data linkages. During live events, this process can be further streamlined by having participants wear name tags, so that independent raters can identify students with no knowledge of their names and minimal introductions during the event. Coloured lanyards or scrubs can be used as a powerful mechanism to identify participants within teams. For example, nurse roles may wear red lanyards/scrubs, while physician roles where blue, etc. This can further be used to link survey data within groups even when it is anonymized (e.g., blue is associated with an individual study ID of 1, red 2, etc.).

Even with such tools, it is often challenging to capture sufficient nuances in complex performance episodes and/or reliability suitable for assessment in dynamic, live environments (36). Use of video recording technology can facilitate this process and enable the development and implementation of more refined grading criteria. For virtual events, Microsoft Teams can be useful for identifying participants; though, it requires participants to log into their accounts. We advise use of the transcript function embedded in these systems, as they are invaluable in helping raters identify speakers to grade more accurately and efficiently. Further, video recording provides a mechanism that enables the use of multiple raters, without the scheduling challenges typically associated with this decision. Use of recordings can enable more thorough assessment of accuracy as well as inter-rater reliability, thus, enhancing rating confidence and quality (36). Video recordings of performance episodes themselves can be used as source material in assessment. For example, it permits videos to be distributed to students so they can review their performance in conjunction with expert feedback, which is superior for student learning compared to traditional feedback methods (37). Further, it can support separate grading for different purposes (e.g., if grading criteria are different for summative relative to formative feedback purposes). Video recording enables greater professional collaboration across schools, as the occurrences can be referred to by each professional identity to evaluate their learners and areas for improvement. For example, two Dutch universities of Applied Sciences used this strategy to assess students’ interprofessional collaboration skills using five interdisciplinary raters spanning expertise in psychology, nursing, educational sciences, physiotherapy and education sciences, and pedagogy (38). Use of this diverse set of raters (made possible by video recording) enabled the study to produce high quality and comprehensive insights into students’ performance as well as the adequacy of the assessment tools and tasks (38). Where available, videos of previously evaluated sessions may be utilized to facilitate rubric development and rater training to reduce the time these take to implement. Use of previous sessions as learning opportunities is crucial when attempting to utilize real time ratings of the event, as preliminary pilot or training sessions utilizing video recordings without rewind/pause functions allow weaknesses of the assessment for live ratings to be identified and resolved prior to implementation (e.g., if the assessment items are too numerous or complex to be reliably assessed in real time). Continuously evaluating findings of these assessments relative to organizational goals is vital to ensure that the program continues to meet institutional needs. This can be accomplished through continued participation of consensus groups through data analysis phases, and the insights of these groups can be leveraged to lead to further improvement in the curriculum, implementation, and assessment practices. Additional tips for selecting appropriate raters and developing grading rubrics for assessment, as well as supporting continuous improvement of programs are discussed in Williams et al. (39).

## Discussion

Interprofessional education is vital to ensuring that future healthcare professional graduates are “team ready” upon graduation. It is necessary to minimize potential negative impacts of stereotypes in the workplace as well as prepare learners to be more equipped to participate in teamwork immediately upon graduation, as their role increasingly transitions to that of a provider rather than learner. Despite significant progress over the last decade, many challenges remain to adequately integrate teamwork and IPE education into the undergraduate space. Scheduling and time constraints remain a significant burden across curriculums to integrate interprofessional learning opportunities and competencies. Further, it has remained a challenge to ensure adequate and accurate representation from various health professionals and retain key faculty throughout implementation of curriculums.

Effective teamwork and IPE should negate or minimize the potential negative impacts of stereotype development, facilitate personal bonding, effective teamwork and collaboration skills across educational boundaries leading to consistent demonstration of positive outcomes across learners. This article has presented several strategies to improve integration of interprofessional education into the undergraduate healthcare education space. These span curriculum design, implementation, and assessment, and have been guided by the collective expertise and experience of our Team FIRST working group that includes administrators, healthcare professionals, educators, and assessment experts.

Pertaining to design, we highlight strategies for developing standardized teamwork training needs analyses for undergraduate professional education. These assessments should utilize objective evaluation methods, such as card sorting tasks (e.g., to assess the how shared mental models are based on how similarly team members group relevant patient- or role-based information), teamwork context surveys (e.g., to assess features of the environment team members perform within, such as hierarchy, information transfer, department interdependence, and task prioritization structures), and team performance simulations (e.g., execution of a code situation which can be assessed by trained observers), to mitigate biases and improve accuracy. Using consensus methods to identify and align these assessments with established competency frameworks, such as those provided by the CIHC, IPEC, and AAMC EPAs ensures a comprehensive approach to evaluating and meeting educational needs.

Regarding implementation, integrating faculty representatives from different disciplines into IPE is essential. This strategy ensures adequate professional representation, appropriateness of the curriculum, and helps mitigate barriers related to coordinating professional school schedules and balancing student ratios. We further expand on strategies to develop faculty with piloting processes, resources, opportunities for curriculum co-creation, and post-activity debriefing sessions. Despite the use of these methods, it is plausible institutions may still encounter barriers due to either resistance to change or lack of interprofessional experience among faculty. Maintenance of the groups formed during consensus meetings associated with the needs analysis and curriculum development are a key source to prevent these barriers from occurring and ameliorate their negative impact during implementation.

Related to assessment, we outline several mechanisms to more effectively develop longitudinal assessment systems. Establishing a comprehensive database that links student names, nicknames, and ID numbers is recommended to improve data linkages and facilitate longitudinal assessment, while we suggest tools like REDCap for efficient and accurate data collection and management, enabling better tracking of student progress and outcomes. We further advocate using video recordings to capture complex performance episodes, which enables the use of multiple raters and more refined grading criteria. These recordings can be used to enhance inter-rater reliability (i.e., consistency between raters) and provide a mechanism to deliver valuable feedback to learners. Videos support separate grading purposes, such as summative and formative assessments, and facilitate professional collaboration across schools.

Notably, we recommend using consensus methods with varied groups of stakeholders across each of these phases of curriculum development, implementation, and assessment. Reaching consensus across stakeholders will be useful to help organizations overcome common barriers to change in healthcare education. We recognize that the resources required for these initiatives may seem daunting; these organizations may find it helpful to engage in digital tools to foster more informal or asynchronous collaboration between these groups to reduce the time commitment required and the likelihood of scheduling conflicts negatively impacting participation. In summary, these strategies aim to create a robust, effective, and sustainable IPE curriculum that enhances collaboration and teamwork among future healthcare professionals. By addressing the key areas of design, implementation, and assessment, this article offers comprehensive guidelines for advancing interprofessional teamwork education. We believe institutions incorporating guidance from this article may offer some relief from existing challenges to IPE and generate an effective teamwork curriculum earlier in undergraduate healthcare education.

## Limitations and future directions

As healthcare systems grow more complex, the coordination of interprofessional education across diverse professions and competencies presents increasing challenges. Due to the variation in existing competency frameworks and the distinct objectives of different organizations, recommending a single, universally acceptable competency framework is currently unfeasible. While institutions are working toward internal consensus on competency standards, curriculum, and assessment practices, further interorganizational, national, and international collaboration is essential to refine frameworks that can be broadly adopted. Such efforts will significantly support the development of competencies and assessments that are rigorously aligned with best practices and demonstrate positive effects on long term outcomes.

## Data Availability

The original contributions presented in the study are included in the article/supplementary material, further inquiries can be directed to the corresponding author.
